# Supplementary material for: Splice-Junction-Based Mapping of Alternative Isoforms in the Human Proteome
Source: Cell Rep. Author manuscript; Available in PMC 2020 Jan 15. (PMC6961840; doi:10.1016/j.celrep.2019.11.026)
Supplement: 3 [file NIHMS1546469-supplement-3.zip › DF2/PXD0010154/AdrenalGland-150-P55196-ADGSGYGSTLPPEKLPYLVELSPDGSDSR.pdf]

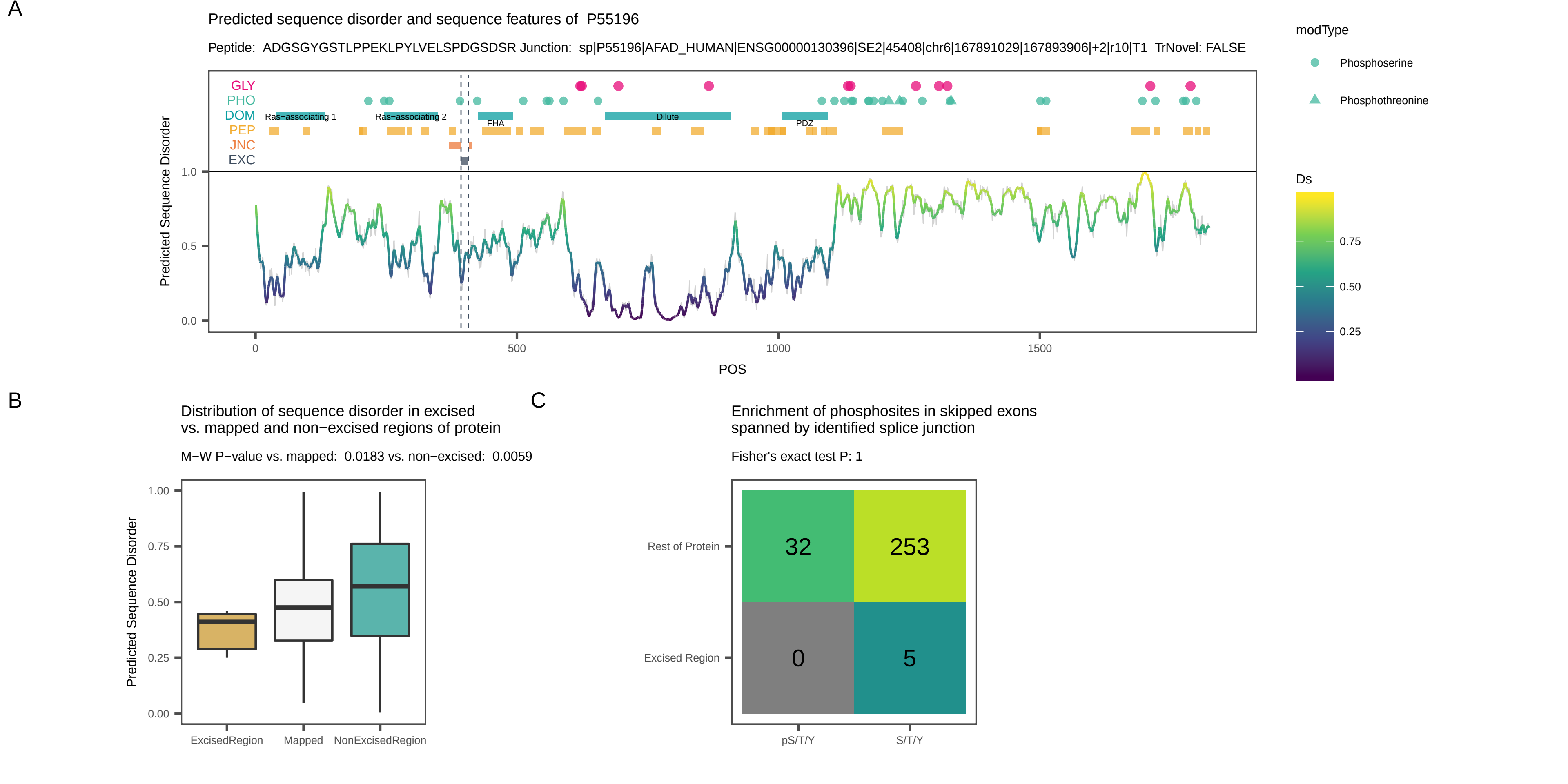
A
Predicted sequence disorder and sequence features of P55196
Peptide: ADGSGYGSTLPPEKLPYLVELSPDGSDSR Junction: sp|P55196|AFAD_HUMAN|ENSG00000130396|SE2|45408|chr6|167891029|167893906|+2|r10|T1 TrNovel: FALSE
GLY
PHO
DOM
PEP
JNC
EXC
Ras−associating 1
Ras−associating 2
FHA
Dilute
PDZ
Predicted Sequence Disorder
POS
modType
Phosphoserine
Phosphothreonine
Ds
B
Distribution of sequence disorder in excised vs. mapped and non−excised regions of protein
M−W P−value vs. mapped: 0.0183 vs. non−excised: 0.0059
Predicted Sequence Disorder
ExcisedRegion
Mapped
NonExcisedRegion
C
Enrichment of phosphosites in skipped exons spanned by identified splice junction
Fisher's exact test P: 1
Rest of Protein
Excised Region
32
253
0
5
pS/T/Y
S/T/Y
